# Understanding recovery of language after stroke: insights from neurovascular MRI studies

**DOI:** 10.3389/flang.2023.1163547

**Published:** 2023-06-05

**Authors:** Maria V. Ivanova, Ioannis Pappas

**Affiliations:** 1 Department of Psychology, University of California, Berkeley, Berkeley, CA, United States; 2 USC Stevens Neuroimaging and Informatics Institute, University of Southern California, Los Angeles, CA, United States

**Keywords:** language, aphasia, perfusion, cerebral blood flow, cerebrovascular reactivity, MRI, post-stroke recovery

## Abstract

Stroke causes a disruption in blood flow to the brain that can lead to profound language impairments. Understanding the mechanisms of language recovery after stroke is crucial for the prognosis and effective rehabilitation of people with aphasia. While the role of injured brain structures and disruptions in functional connectivity have been extensively explored, the relationship between neurovascular measures and language recovery in both early and later stages has not received sufficient attention in the field. Fully functioning healthy brain tissue requires oxygen and nutrients to be delivered promptly via its blood supply. Persistent decreases in blood flow after a stroke to the remaining non-lesioned tissue have been shown to contribute to poor language recovery. The goal of the current paper is to critically examine stroke studies looking at the relationship between different neurovascular measures and language deficits and mechanisms of language recovery via changes in neurovascular metrics. Measures of perfusion or cerebral blood flow (CBF) and cerebrovascular reactivity (CVR) provide complementary approaches to understanding neurovascular mechanisms post stroke by capturing both cerebral metabolic demands and mechanical vascular properties. While CBF measures indicate the amount of blood delivered to a certain region and serve as a proxy for metabolic demands of that area, CVR indices reflect the ability of the vasculature to recruit blood flow in response to a shortage of oxygen, such as when one is holding their breath. Increases in CBF during recovery beyond the site of the lesion have been shown to promote language gains. Similarly, CVR changes, when collateral vessels are recruited to help reorganize the flow of blood in hypoperfused regions, have been related to functional recovery post stroke. In the current review, we highlight the main findings in the literature investigating neurovascular changes in stroke recovery with a particular emphasis on how language abilities can be affected by changes in CBF and CVR. We conclude by summarizing existing methodological challenges and knowledge gaps that need to be addressed in future work in this area, outlining a promising avenue of research.

## 1. Introduction

Healthy brain tissue requires rapid delivery of oxygen and nutrients via its blood supply for aerobic metabolism underlying neural activity to take place (Buxton et al., 2004; Newberg et al., 2005). This makes different measures of blood flow an important indicator of tissue status and functionality. Specifically, cerebral blood flow (CBF) or perfusion indicates the amount of blood being delivered to the tissue and serves as a proxy for the metabolic demands of the brain. Complimentary to CBF, cerebrovascular reactivity (CVR) is a dynamic measure of blood flow. It provides a measure of changes in blood flow in response to a challenge that changes arterial CO_2_. Increased arterial CO_2_ leads to dilation of the arteries and recruitment of collateral arteries, so that oxygen supply to the brain tissue remains constant. CVR specifically measures this ability of the vascular system to compensate for increasing demands. Thus, instead of measuring a static value of CBF, CVR provides researchers the ability to see whether blood flow can change in compromised blood vessels as well as in collateral arteries (Fisher and Mikulis, 2021; Sleight et al., 2021). Together, measures of CBF and CVR offer insights into the functionality and health status of brain areas by capturing both cerebral metabolic demands and mechanical vascular properties. The importance of this complementary information becomes particularly evident in clinical populations when these measures can be acutely altered and subsequently change during recovery.

Blood flow in the brain is disrupted in many disorders, one of which is stroke. In stroke, decreased blood flow to brain areas causes cell death and permanent tissue damage due to the lack of oxygen (Markus, 2004). However, a reduction in CBF can also be observed beyond this core lesion site, in both surrounding and more distal brain areas (Demeestere et al., 2020). The most notable is the penumbra after ischemic stroke, the band of tissue that surrounds the core ischemic lesion. Penumbral tissue contains electrically unexcitable but viable cells, which can regain their function if blood flow is promptly restored (Markus, 2004). In animal models, it was shown that brain tissue needs to be perfused at only 10% of normal levels to survive, and at least at 30–50% for neuronal function (i.e., electrical signaling) to continue (Astrup et al., 1977; Sekhon et al., 1994). These thresholds are likely even higher for humans, though that remains to be confirmed (Hillis, 2007). Beyond perilesional tissue, altered perfusion after stroke has been found in distal areas as well (Mimura et al., 1998; e.g., Brumm et al., 2011; Thompson et al., 2017; Ivanova et al., in press); this could be due to a number of factors, including diaschisis (Carrera and Tononi, 2014). While CBF tracks metabolism in brain regions that survived the stroke insult, it does not capture how spared regions might be supplied via collateral flow. In turn, measures of CVR play an important role in understanding the brain’s ability to supply blood to these spared functional areas (Krishnamurthy et al., 2021). CVR can provide evidence for blood flow impairment in compromised blood vessels as well as collateral flow by quantifying effective blood flow changes in non-compromised tissue (Hoiland et al., 2019). This can lead to additional insights into post stroke recovery that can assess overall health of the spared regions.

The role of injured brain structures and disrupted functional connectivity in post stroke language deficits have been extensively explored (Pustina et al., 2017; Ramsey et al., 2017; Siegel et al., 2018; Kristinsson et al., 2021;Wilson et al., 2023). At the same time the relationship between neurovascular measures and language recovery in both early and later stages has not received sufficient attention in the field. Lower perfusion may impede normal neuronal functioning (Feeney and Baron, 1986; Powers et al., 1987; Girouard and Iadecola, 2006) and, consequently, a sustained decrease in CBF after a stroke to the remaining spared tissue has been shown to contribute to poor language recovery (Boukrina et al., 2019; e.g., Abbott et al., 2021; Ivanova et al., in press). A post stroke reduction in CVR in spared tissue indicates vascular changes and an inadequate blood supply to these regions, which could also negatively affect recovery. Furthermore, tracking changes in CVR over the course of stroke recovery can help to determine whether new collaterals emerge and possibly promote functional reorganization of the language system. Specifically in the context of aphasia, it remains to be answered how neurovascular changes after stroke in different brain regions relate to language outcomes and whether a combination of CBF and CVR measures can provide additional insights into the mechanisms of post stroke recovery.

The goal of the current paper is to critically examine stroke studies looking at the relationship between different neurovascular measures and language deficits and the mechanisms of language recovery via changes in neurovascular measures. In this paper, we review previous work related to CBF and CVR in different brain regions post stroke. We also discuss how these neurovascular measures relate to language and cognitive performance, particularly in those individuals with aphasia. We conclude by outlining existing methodological challenges and knowledge gaps that need to be addressed in future work in this area, outlining promising avenues of language research.

## 2. Neurovascular changes in stroke

### 2.1. Cerebral blood flow

Initial studies looking at cerebral perfusion and its relationship to language recovery post stroke have mostly relied on perfusion weighted imaging (PWI) methods that require the administration of a contrast agent (typically gadolinium-based) (Essig et al., 2013). PWI tracks the delayed arrival of the agent bolus to different brain regions and from these data maps of cerebral blood volume and flow are derived. Hillis and colleagues have extensively looked at cohorts of aphasia patients in acute stage with the use of PWI and found that aphasia is linked to cortical hypoperfusion (Hillis et al., 2002; Hillis, 2007). At the same time, reperfusion of critical brain areas early on has been linked to recovery of language function. For example, reperfusion of Wernicke’s area within the first days post stroke has been associated with improvement in language comprehension (Hillis and Heidler, 2002). Today the use of PWI is limited to the clinical evaluation of perfusion in acute cerebral ischemia and brain tumors due to the administration of exogenous contrast (Essig et al., 2013), while current research studies in aphasia are exclusively based on magnetic resonance imaging (MRI) methods without exogenous contrast.

Arterial Spin Labeling (ASL) is the primary MRI method that can non-invasively quantify blood flow in the brain by using magnetized blood as an endogenous contrast agent. ASL starts by inverting magnetization of incoming arterial blood at the neck level via radiofrequency pulses (“tagging”), and after a certain delay, “labeled” images of the brain are acquired containing signals from the magnetized blood. “Control” images are also acquired without prior tagging and the difference between the two provides a metric of labeled blood from the arteries of the brain or CBF (Alsop et al., 2015). CBF or perfusion is defined as the blood volume that flows per unit of brain tissue per unit of time and is typically expressed in units of ml blood/100 g tissue/min (Alsop et al., 2015; Lindner et al., 2023). In human adults, normal CBF in different gray matter regions ranges from 35 to 80 mL/100 g/min depending on factors such as age, sex, diet, sleep, psychological state, cardiovascular fitness, and health status (Leenders et al., 1990; Parkes et al., 2004; Clement et al., 2018; Joris et al., 2018; Abbott et al., 2021). Thus, CBF directly measures the amount of blood being delivered to different brain regions and serves as a proxy for the metabolic demands of these areas. Following stroke changes in CBF are observed both acutely and chronically in many different brain areas.

#### 2.1.1. Perilesional tissue

Perilesional tissue is a part of the brain directly adjacent to the lesioned regions that are permanently damaged. Typically, perilesional areas are defined as a band of 5 or 10mm around the lesion, though the specific spatial demarcations vary significantly across studies (see below for more on this). Research on acute stroke has demonstrated that CBF is often disrupted in perilesional tissue (Chalela et al., 2000; Demeestere et al., 2020). Further, decreased perfusion (also called hypoperfusion) in perilesional areas persists in the chronic phase relative to homologous areas in the contralesional hemisphere and similar regions in age-matched healthy controls (Fridriksson et al., 2002; Brumm et al., 2011; Richardson et al., 2011; Thompson et al., 2017; Boukrina et al., 2019). Interestingly, Richardson et al. (2011) demonstrated a strong relationship between infarct size and reduction in perilesional perfusion (defined as a 3–8mm band around the original infarct) relative to the right hemisphere homologous regions in chronic ischemic stroke (*n* = 17). These findings imply that individuals with larger infarcts might be doubly impacted by both larger structural lesions and larger areas of hypoperfusion surrounding the lesion (but see Abbott et al., 2021 for contradictory findings). A recent study argued for an individualized approach when determining hypoperfusion in perilesional tissue where abnormal values are those below 1.5 standard deviations of the average right hemisphere CBF (Abbott et al., 2021). Abbott et al. (2021) used the proposed individualized criterion for determining functionally compromised brain tissue, in a small cohort of individuals with chronic aphasia (n = 6). The authors observed the most marked changes within a 0–3mm band around the lesion, with perfusion returning to normal values outside of that ring.

In stroke, the extent of hypoperfusion of perilesional tissue has been associated with severity of cognitive and language impairment (Vallar et al., 1988; Fridriksson et al., 2002; Brumm et al., 2011; Motta et al., 2015; Robson et al., 2017; Thompson et al., 2017) and motor deficits (e.g., Wiest et al., 2014). Fridriksson et al. (2002) in a cohort of 9 participants with aphasia showed that the extent of hypoperfusion surrounding the lesion (but not lesion size) was related to aphasia severity at 1 day and at 1 month post onset. These results are similar to a number of earlier perfusion studies done with single photon emission computerized tomography (Vallar et al., 1988;Nakagawa et al., 2005). More recently Motta et al. (2015) showed a statistical association between reperfusion of perilesional tissue (defined by a diffusion-perfusion mismatch based on PWI and diffusion MRI within 24 h of onset) and cognitive outcomes at 2 weeks post onset in a large group of individuals with acute ischemic stroke (n = 38). In their study, a reduction in hypoperfused perilesional tissue volume was related to improvement in cognitive abilities (naming ability for left hemisphere patients and line cancelation for right hemisphere patients), further emphasizing the critical role that perilesional tissue plays in stroke recovery.

Specifically with regards to language outcomes in chronic stroke, Brumm et al. (2011) demonstrated that in three chronic ischemic stroke survivors, lower perfusion in the ischemic penumbra was associated with more severe aphasia. Thompson et al. (2017) observed a positive relationship between perfusion levels in the perilesional tissue (0–6mm band) and naming, sentence comprehension and production abilities in a large group of individuals with chronic aphasia following ischemic stroke (*n* = 35). In another large sample of chronic post stroke aphasia (*n* = 43) a relationship between perilesional perfusion (0–5mm band) and fluency, naming, and overall aphasia severity was also observed, although this relationship was not as strong as for some of the language areas (parietal and temporal) (Ivanova et al., in press). More distant perilesional bands (5–10 mm and 10–15 mm) did not show such a relationship. Thus, out of all the perilesional bands that the authors investigated, only perfusion in tissue directly adjacent to the lesion was pertinent for language outcomes. More distant perilesional areas (5–10 mm and 10–15 mm) likely encompass different cytoarchitectonic areas that are unable to functionally compensate for the lesioned tissue. In another study in twelve individuals with chronic Wernicke’s aphasia, a strong association between language abilities and CBF in perilesional areas was demonstrated (Robson et al., 2017). However, in this study the definition of perilesional tissue was based on hypoperfusion values rather than a specific distance from the core lesion, making it harder to dissociate effects seen in perilesional areas from that of areas more distant from the lesion.

Not all studies find a systematic association between perilesional perfusion and functional outcomes. Fridriksson and colleagues were unable to demonstrate a significant relationship between pretreatment levels of CBF or change in CBF in perilesional cortex (3–15 mm around the lesion) and improvement in naming ability following anomia treatment in chronic stroke (n = 30) (Fridriksson et al., 2012). Similarly, perfusion of perilesional tissue (0–5 mm) was not significantly associated with current reading deficits (although there was a positive trend) or recovery of reading abilities in a small longitudinal study (*n* = 15) (Boukrina et al., 2019). Likewise, levels of perilesional perfusion did not change following language therapy and were not predictive of treatment outcomes in a large group of people with chronic aphasia (*n* = 45) (Walenski et al., 2022).

To summarize, studies that measured CBF acutely after stroke found that failure to restore sufficient blood flow to perilesional tissue results in persistent cognitive and language deficits observed at the acute as well as chronic stages. Although at the chronic phase, the associations between levels of perilesional perfusion and language abilities were less consistent. Conflicting findings in chronic stroke might be partially due to varying definitions of perilesional tissues, as currently there is no consensus on how far from the infarcted region should the tissue still be considered perilesional and whether partial volume effects potentially dilute the functional relationships (for a related argument see Abbott et al., 2021; also, for more on this see Section 3. Methodological Considerations below). Additionally, it is not clear whether right hemisphere areas that could occasionally fall into expanding perilesional masks are systematically excluded. So far, the exclusion of right hemisphere areas from perilesional masks was only explicitly reported by Ivanova et al. (in press). Finally, it remains unknown to what extent changes in perilesional perfusion in chronic stroke can promote continuous language recovery. Possibly by this late in their recovery, changes in the perilesional space have already taken place (Lee and Donkelaar, 1995; Nudo, 2013). It may also be the case that perilesional perfusion is more relevant in cases of motor recovery, as motor function is more modular and spatially restricted, i.e., more distant areas are not capable of taking on functions of the motor cortex (Nudo, 1999). In all, while overall the evidence is in favor of perilesional perfusion being reduced and related to functional outcomes, the boundary for this underperforming tissue that may be crucial for recovery still needs to be determined along with the specific timeframe when reperfusion of that tissue is particularly critical for optimal language recovery.

#### 2.1.2. Distant ipsilateral areas

Disruption of perfusion in stroke goes beyond perilesional areas and has been observed in ipsilateral areas distant from the lesion in chronic stroke (Mimura et al., 1998; Richardson et al., 2011; Abbott et al., 2021). In the first study of perfusion in aphasia, Mimura et al. (1998) showed reduced CBF in the left hemisphere relative to the right hemisphere in subacute stroke (*n* = 20) and lower values relative to controls in chronic stroke (*n* = 16). Further, those individuals with good language recovery showed higher left hemisphere perfusion and more pronounced changes in left hemisphere perfusion in the first year post stroke, with similar associations with functional outcomes observed in the chronic stage as well. However, this early study was restricted to one slice of single-photon emission computed tomography data, limiting its generalizability and spatial accuracy.

Subsequently, several single case and small group studies in aphasia have documented abnormal vascular physiology and lowered perfusion in the left hemisphere. Love et al. (2002) demonstrated that lower perfusion in the left supramarginal and angular gyri in a patient with chronic stroke was associated with functional impairment in reading ability, while no abnormalities in those areas were observed on structural scans. Brumm et al. (2011) documented both increased transit delays and decreased CBF in three individuals with chronic stroke. As expected, hypoperfusion was most notable in perilesional areas, but also documented in distant regions from the lesion. However, the authors did not investigate the relationship between functional outcomes and CBF levels in these distant left hemisphere regions. In one study focused on acute stroke, it was shown that language recovery was driven by reperfusion of frontal and temporal regions in the left hemisphere critical for language, but this effect was observed only in one patient out of five (Jarso et al., 2013).

A recent comprehensive study of perfusion in chronic aphasia (Thompson et al., 2017) showed a decrease in perfusion in several areas in the left hemisphere compared to healthy controls (and interestingly an increase in the superior frontal gyrus). However, these differences were not associated with functional language outcomes. Based on the findings of this study, it appears that left hemisphere regions in the distribution of the middle cerebral artery are hypoperfused, while regions in the anterior cerebral artery can be hyperperfused. A recent small cohort study employing individualized perfusion cutoffs based on right hemisphere perfusion values demonstrated a strong relationship between hypoperfusion of regions in the left posterior temporal and inferior parietal areas and general language ability and auditory comprehension (Abbott et al., 2021). Further, another large study in chronic stroke showed that residual perfusion in the left temporal lobe (most prominently in the posterior part of both superior and middle temporal gyri) and in the inferior parietal areas (supramarginal gyrus) was significantly related to different language abilities (Ivanova et al., in press). Levels of CBF in the temporal areas contributed most strongly to auditory comprehension and naming abilities, while CBF in parietal regions—to fluency and repetition. This relationship was present even when direct lesion damage to these areas was accounted for. These findings indicate that spared blood flow in those areas is important for supporting language function. Importantly, in the same study no relationship with language was observed for perfusion levels in control areas within the ipsilesional hemisphere, underscoring the anatomical specificity of the observed results (Ivanova et al., in press). Interestingly, a recent study showed that cortical hypoperfusion in the left hemisphere was more likely a determining factor of language deficits in subcortical aphasia rather than direct effect of the lesion in the basal ganglia (*n* = 19) (Celebi et al., 2022), replicating an effect observed earlier in acute subcortical aphasia (Hillis et al., 2002). However, another study did not find cortical hypoperfusion to be a contributing factor to the severity of acute subcortical aphasia (Sharif et al., 2022). The discrepancies in the results are likely due to varying methods used to assess cortical perfusion, with the latter study with null effects relying on hyperintense vessels on FLAIR scans to estimate regions of decreased blood flow.

Perfusion levels have been identified as a prognostic factor in stroke recovery, with initially higher CBF predictive of better treatment outcomes in aphasia (Thompson et al., 2010; Fridriksson et al., 2012; Boukrina et al., 2019). Thompson et al. (2010) found an association between baseline perfusion levels and propensity for upregulation of activity (as measured by the blood-oxygen-level dependent (BOLD) response in a task-based functional MRI). Specifically, baseline perfusion was higher (i.e., closer to normal levels) in cortical regions that showed upregulation of neural activity in a small group of six patients who underwent treatment for agrammatism, demonstrating that areas that are better perfused may have more treatment potential. Fridriksson et al. (2012) found a similar pattern in 30 patients who received treatment for anomia; pretreatment perfusion levels in undamaged regions within the left hemisphere language network (excluding infarcted and perilesional regions) predicted patients’ naming accuracy post-treatment. These findings once again suggest that higher baseline CBF may be related to the potential for a better treatment outcome. Similarly in a recent longitudinal observational study (Boukrina et al., 2019), increased perfusion of intact areas within the reading circuit in the left hemisphere in the subacute stage predicted phonological and reading ability (but not semantic or orthographic abilities) at 6 months post stroke in 15 patients. At the same time, as noted in the previous section, baseline perfusion levels in the perilesional tissue in the last two studies (Fridriksson et al., 2012; Boukrina et al., 2019) did not predict language recovery.

Cumulatively, the literature to date indicates that while perfusion in stroke will often be delayed and lowered in distant ipsilateral areas, there seems to be substantial individual variability, reflecting the influence of factors such as stroke type and severity, lesion site and size, time post-onset, age, as well as vascular and general brain health. Existing evidence suggests that hypoperfusion in language-salient left hemisphere regions is at least partially contributing to persistent language deficits, however, the specific and differential role of perfusion levels in different regions of the language network remains to be established.

#### 2.1.3. Right hemisphere areas

In contrast to perfusion of ipsilateral areas, hypo- or hyperperfusion of homologous contralateral areas has not been consistently associated with functional outcomes in stroke. In individuals with good motor recovery following sensorimotor stroke, perfusion levels actually decreased in the contralesional hemisphere (Wiest et al., 2014). With regards to language, Mimura et al. (1998) showed that language recovery in the first year post stroke was independent of changes in perfusion in the right hemisphere. However, in the same study those individuals with chronic stroke (around 7 years post-onset) and good language recovery had higher right hemisphere perfusion values compared to those with poor recovery and showed similar right hemisphere CBF to healthy controls. However, unlike that for regions in the left hemisphere, no association with language outcomes and right hemisphere perfusion was observed (Mimura et al., 1998). Still another functional MRI study (Peck et al., 2004), showed that the time-to-peak values of the hemodynamic response in pre-supplementary motor area, Broca’s area homolog, and motor and auditory cortices in the right hemisphere decreased following improvement in naming ability in three patients with chronic post stroke non-fluent aphasia, substantiating the compensatory role of the right hemisphere in recovery. The authors concluded that the results reflect increased speed of language processing.

However, a recent study in chronic stroke (Thompson et al., 2017) demonstrated that individuals with aphasia had increased perfusion in regions of the right hemisphere compared to the left hemisphere (once lesion was accounted for) and also compared to the right hemisphere perfusion in age-matched healthy controls (although this difference was only of borderline significance). However, the increased perfusion in the right hemisphere was not related to functional outcomes. The authors hypothesized that hyperperfusion of the right hemisphere likely indicates autoregulatory changes in blood flow following stroke and/or increases in general cognitive effort, rather than maladaptive language processing. Similarly, Ivanova et al. (in press) showed interhemispheric differences in perfusion in chronic aphasia, with greater CBF observed in the right compared to the left hemisphere. They observed no association between perfusion levels in right hemisphere regions and language outcomes. Boukrina et al. (2019) also showed increased perfusion in the right hemisphere reading network relative to the left hemisphere in a large group with subacute stroke (*n* = 31). Interestingly, higher perfusion in the right hemisphere was associated with lower word reading accuracy both in the subacute and the chronic stages, but not related to performance on other language tasks involving phonological, semantic, and orthographic processing.

Thus, there is inconclusive evidence on whether reperfusion of right hemisphere areas to normal levels can support and promote recovery. It is more likely that an increase in contralateral perfusion in the late chronic stages in stroke reflects largely vascular changes, rather than underlying reorganization of function. This observation likely undermines the proposed individualized approach (Abbott et al., 2021) for determining tissue perfusion based on contralateral perfusion values.

### 2.2. Cerebrovascular reactivity

In stroke, CBF measurements can be influenced by both demand (how much the particular area is active) and supply constraints (how much blood can be delivered to that tissue). Following a stroke, blood flow via collateral pathways may be able to maintain functioning tissue in areas beyond an occlusion that cannot be captured by CBF measurements (Gabriel-Salazar et al., 2018). In particular, ASL methods capture CBF by measuring magnetized blood circulating in brain tissue ~2–3 s after delivery. Technical factors, the most important of which is relaxation of the tagged blood, makes it difficult to observe delivery longer than approximately 4 s. However, in certain cases (such as occlusion), blood can reach the region of interest via collateral pathways and at a time scale beyond the ASL technical limits. Thus, mapping these compromised territories is beyond the limits of ASL and requires other MRI modalities. Characterization of collateral blood flow is of particular interest in understanding residual function. Brain regions that appear hypoperfused on ASL scans can show different levels of residual function depending on whether they can recruit additional blood via either dilation or collateral blood flow. CVR specifically addresses this need, and it is a method to assess the ability of the brain’s vasculature to change blood flow in response to a challenge. CVR mapping involves measuring the brain’s response to a global vasodilator, typically a hypercapnic challenge such as CO_2_ gas inspiration or breath holding. During these challenges, there is an elevated arterial CO_2_ level due to CO2 inhalation or due to breath holds that in turn leads to vasodilation (Liu et al., 2019). Thus, CVR reflects changes of blood flow in response to the stimulus via vasodilation. One can then use CVR to see how much blood flow changes in different vessels and collateral pathways (Sam et al., 2014; Sobczyk et al., 2021a).

CVR is typically measured by regressing BOLD signals against the measured CO_2_.traces obtained during the challenge, thus measuring the changes in BOLD in response to changes in CO_2_. In animal models, CVR was decreased after an ischemic injury not only in permanently damaged regions but also in tissue that recovered (Olah et al., 2000). This result indicates that a prolonged disturbance of CVR can occur despite recovery of energy metabolism in tissue. In humans, there are few studies looking at CVR changes post stroke, mostly due to the experimental difficulties of obtaining such measurements in clinical populations. In one study examining CVR in stroke patients using resting-state BOLD signals, CVR was decreased in the core of the lesion but increased in the perilesional regions (Taneja et al., 2019). The authors claim that it might reflect a compensatory mechanism whereby blood vessels in these regions are dilated to preserve CBF. CVR to a vasoactive stimulus can also reveal collateral pathways via a blood “steal” phenomenon, whereby regions that are fed in parallel from a common artery are in competition. During large increases in arterial CO_2_, the blood flow in compromised pathways with reduced vascular reserve is not sufficiently increased and is redistributed to vascular beds with more robust vasodilation. This allows for the mapping of collateral vessels with non-compromised reserves that can still provide blood to regions of interest (Duffin et al., 2017; Sobczyk et al., 2021a). Examination of CVR in stroke patients have revealed hemispheric “steal” (Fisher et al., 2018) although more systematic investigations are required.

CVR mapping at the acute and subacute phases can reveal the extent of vascular changes including the range of collateral blood supply to the areas outside the lesion. Understanding CVR changes over the course of stroke recovery and how these changes relate to language recovery is a vastly understudied field (Krishnamurthy et al., 2021). Preliminary data from the aging and dementia literature suggest a link between reduced CVR and cognitive decline (Richiardi et al., 2015). A systematic review of CVR studies found decreased CVR in frontal regions in patients with mild cognitive impairment and Alzheimer’s patients (Sleight et al., 2021). However, to date there are no studies looking explicitly at the link between language or cognitive post-stroke deficits and CVR. Studies measuring CVR in stroke patients have suggested that CVR in the perilesional tissue and surrounding areas was not different between controls and patients both at 2- and 4-months post stroke (Krainik et al., 2005; Geranmayeh et al., 2015). In another study, the authors used CVR to account for hemodynamic differences in measures of language-related fMRI tasks during recovery in aphasia. The authors did not observe differences between patients and controls thus they argue that language recovery could not be attributed to hemodynamic differences (van Oers et al., 2010). The discrepancy between the previous literature and the stroke literature is potentially due to the differences in the methods these studies employed and task compliance. More studies in the future should systematically examine changes in CVR post stroke and their relationship to language recovery. All in all, given the previous evidence and the complementary data from the aging literature, one hypothesis that remains to be tested is that reduction in CVR in the perilesional and related language areas at the acute phase leads to worse language outcome as a result of failure to establish collateral flow.

### 2.3. Neurovascular changes following treatment-induced recovery

Levels of CBF (and possibly CVR) post stroke are positively related to residual language abilities. In turn, improvements in blood flow post stroke can happen spontaneously, especially in the acute and sub-acute stages of recovery, or through targeted interventions. Different interventions, including speech-language therapies (Brady et al., 2016), noninvasive brain stimulations (Breining and Sebastian, 2020; Zhang et al., 2021), and pharmacological treatments (Stockbridge, 2022), have demonstrated positive effects on language outcomes in post-stroke aphasia (Berube and Hillis, 2019). These interventions have also shown to affect neurovascular measures. Therefore, it is reasonable to assume, that the mechanisms of spontaneous recovery in the context of CBF and CVR as described previously and those of treatment-induced language recovery might be similar (Cassidy and Kramer, 2017).

Still the findings regarding neurovascular changes post treatment in the stroke population remain very limited to non-existent, with no studies to date focusing specifically on language outcomes. However, one can look to studies in other populations for insights into possible neurophysiological mechanisms behind observed behavioral gains. For example, in healthy participants it has been shown that Transcranial Magnetic Stimulation (TMS) increases CBF as measured by ASL MRI (Orosz et al., 2012; Gratton et al., 2014). In one study in stroke, increases in perfusion following repetitive TMS were associated with motor outcomes (Takekawa et al., 2014). Similarly, some behavioral interventions in healthy participants, such as physical exercise, have been shown to augment CBF and CVR in specific brain areas that can potentially facilitate language and cognitive recovery post stroke (Macintosh et al., 2014; Steventon et al., 2020). Only one study in stroke looked specifically at changes in CBF as a result of a 19-week exercise training and found increased CBF in the medial temporal lobe (Moore et al., 2015). For pharmacological interventions, beyond the acute stage, there has been limited research on the effects of drugs on neurovascular measures although past studies have shown that levodopa can increase CBF in other clinical populations (Kobari et al., 1992).

All in all, carefully designed interventions have the potential to augment language recovery and an increase in CBF and/or CVR might be one of the brain mechanisms behind observed treatment effects. Different facets of therapies need to be taken into consideration when interpreting treatment-induced behavioral and brain changes. In addition, interventions need to consider the heterogeneity of the populations (whether they are targeting chronic vs. acute stroke populations), the baseline severity, along with other clinical characteristics. Further, participants with already compromised CBF or CVR might have reduced capacity for reorganization and show limited benefits from such interventions. Thus, therapies might need to take into consideration neurovascular measures at baseline and potentially stratify participants by baseline CBF or CVR. Although more research is needed, the mechanisms by which these interventions work likely overlap with those of spontaneous recovery (Cassidy and Kramer, 2017). Therefore, neurovascular measures have the potential to both provide critical insights into mechanisms of recovery and explain differential gains made in treatment by different patient subgroups.

## 3. Methodological considerations

### 3.1. Cerebral blood flow

The publication of a consensus paper on the clinical implementation of ASL in 2015 provided guidelines regarding “best practices” of ASL imaging (Alsop et al., 2015). The authors recommend pseudocontinuous ASL (pCASL), as the primary workhorse sequence for ASL imaging. Continuous ASL is a type of imaging that uses a continuous labeling scheme, meaning that the blood is typically magnetized for 1–3 s through a single labeling plane by a continuous RF power (Alsop et al., 2015). Compared to continuous ASL imaging methods, pCASL uses a train of radiofrequency (RF) and gradient pulses and this has been shown to improve labeling efficiency (Dai et al., 2008). The authors also recommend specific imaging parameters crucial for optimal ASL image acquisition. These parameters are the labeling duration, which is the duration for which the blood is tagged by RF pulses, and the post labeling delay (PLD), which is the time waiting for the magnetized blood to reach the brain. The labeling duration, a parameter that depends on the T_1_ relaxation of the blood, according to the authors should be set to 1,800 ms at 3 T. In turn, the PLD needs to be carefully selected to keep a balance between signal-to-noise ratio (due to T1 decay longer PLDs inevitably have lower signal-to-noise ratios) and complete bolus delivery, which varies depending on the target population. Thus, the authors recommend a PLD of 2000ms for older adults and clinical populations of interest (Alsop et al., 2015). A recent consensus paper on the applications of ASL in the clinical setting also proposed the use of single delay ASL sequences with sufficiently long PLDs that are tailored to the age of the population (e.g., 2,000ms for older populations or populations with impaired cerebrovascular health) (Lindner et al., 2023).

When the time for the blood to reach the brain tissue from the labeling position is abnormally high (for example, in regions downstream to arterial occlusion), this can affect CBF values. In this case, the use of multi-delay methods (multi-PLD) is recommended. These can be quantified based on modified PCASL acquisition methods with scans of different PLDs (Wang et al., 2013). Multi-PLD ASL scans thereby permit the calculation of an arterial transit time (ATT) map via weighted combination of the PLDs as well as CBF. However, caution needs to be taken when applying these sequences to clinical populations due to their long acquisition time and lower signal-to-noise ratio at long delays. Toward this direction, multi-PLD sequences are now becoming increasingly mature with the combination of gradient and spin echo (GRASE) readouts for faster acquisition and such sequences are now being successfully applied to quantify CBF and ATT in stroke populations (Wang et al., 2013). The longer total acquisition time required to collect multiple PLDs can be offset somewhat by efficient approaches such as time-encoded pCASL using Hadamard encoding (van Osch et al., 2018).

Further ASL imaging acquisition choices relate to readout, reconstruction, and preprocessing techniques, all of which can have a significant impact on the CBF data obtained. The 2015 consensus paper (Alsop et al., 2015) proposed 3D GRASE or 3D stack-of-spirals for readout. Three-dimensional encoding is preferred because the transit delay is constant over the entire brain, unlike a multi-slice 2D EPI scheme. Since then, additional recommendations have been proposed that include advanced readout techniques that use varying flip angles or advanced spiral designs, all 3D. These techniques construct more consistent perfusion signals in the readout phase that can reduce signal dropout and mitigate spatial blurring (Hernandez-Garcia et al., 2022). In addition, because ASL MRI is prone to motion and other physiological noise, it is recommended that motion correction is used alongside certain noise reduction techniques such as outlier cleaning or noise component removal (Wang, 2012; Carone et al., 2019). Finally, because ASL spatial resolution is coarse compared to structural scans (usually, 2–4 mm in-plane), some authors recommend partial volume correction methods, although validation of these methods is still ongoing and its benefits for clinical populations (particularly in the presence of large stroke lesions that impact segmentation algorithms) remain to be established (Chappell et al., 2021).

### 3.2. Cerebrovascular reactivity

For quantitative measurement of CVR, CO_2_ gas inhalation is considered the gold standard (Sobczyk et al., 2021a). However, gas inhalation is quite laborious and impractical in the clinical setting. Thus, exhalation methods based on breathing tasks have been implemented and yield semi-quantitative maps of CVR. In these experiments, participants are asked to do a cued breathing task (“breath in”, “hold your breath”, “breath out”) while an expired CO_2_ trace is being recorded (Chen and Gauthier, 2021). The end-tidal CO_2_ is then regressed against the fMRI response to produce a CVR map. Multiple studies have used breathing tasks to show robust CVR responses in healthy populations (Sobczyk et al., 2021b) as well as abnormal CVR patterns in different clinical conditions such as Moyamoya disease (Stickland et al., 2021). Although a breathing task is more practical to deploy than a gas inhalation setup, there are a variety of factors that can affect the measured CVR response. In breathing tasks, task compliance is highly variable among individuals, thus washing away group effects. Another potential limitation is that the increased arterial CO_2_ from breath holding may be insufficient to reveal all regions vulnerable to blood steal (Sobczyk et al., 2021a). Finally, due to the nature of breathing, breathing tasks can induce motion in the obtained fMRI signal. Task-free (“resting state”) fMRI methods have been proposed as alternatives whereby resting-state BOLD fMRI is recorded alongside the CO_2_ tracer. In resting-state CVR methods, the amplitude of changes in CO_2_ is smaller compared to breathing tasks and different across subjects making the CVR modeling even more challenging. In addition, further validation, and reproducibility of these methods especially in older subjects and patients is required (Pinto et al., 2021), not least because of the possibility that the natural fluctuations in arterial CO_2_ may be insufficient to reveal abnormal flow regions, as noted above for breath holding. Given all the considerations outlined above, a breathing task derived CVR provides a compromise between sensitivity and quantification, while being a practical solution for obtaining CVR responses in special populations such as stroke patients. Due to the smaller magnitude and larger variability of the responses, researchers should try and enhance task compliance (perhaps using visual or other cues, and/or using a chest belt) and increase the amount of data obtained to boost signal-to-noise ratio.

## 4. Knowledge gaps and future directions

Language recovery following stroke relies on preserved brain areas (Saur et al., 2006; Kiran and Thompson, 2019), with recent meta-analyses indicating stronger evidence for left hemisphere involvement in recovery (Stefaniak et al., 2021; Wilson and Schneck, 2021). Yet, these studies rarely consider the amount of remaining blood flow to these regions, simply viewing all regions that appear non-damaged on structural scans as preserved. Based on the neurovascular studies reviewed above these regions in some individuals might be functioning suboptimally due to decreased CBF and/or CVR (Geranmayeh et al., 2015; Thompson et al., 2017; Boukrina et al., 2019; Ivanova et al., in press). Regions that are structurally preserved but sub-optimally perfused may hinder language recovery (Thompson et al., 2010, 2017; Fridriksson et al., 2012). The limited number of studies available point to the relationship between levels of perilesional perfusion and cognitive/language outcomes in the acute/subacute phases, and perfusion in temporoparietal left hemisphere regions and language functioning in chronic stages of recovery. At the same time, perfusion studies generally do not find a relationship between neurovascular measures in the right hemisphere and language outcomes, supporting the emerging hypothesis that the right hemisphere does not play a central role in language recovery after a left (dominant) hemisphere stroke (Wilson and Schneck, 2021). Of particular importance are the recent findings that perfusion metrics from regions in the left hemisphere contribute to language outcomes beyond what can be explained by structural damage alone (Ivanova et al., in press). Further, another study has shown that combining multimodal imaging measures, including perfusion, provided greater accuracy for determining deficits in aphasia compared to a single neuroimaging modality (Kristinsson et al., 2021). Together these observations underscore the importance of incorporating neurovascular metrics into the study of the neural mechanisms of post stroke cognitive and language deficits.

Unfortunately, the findings to date do remain mixed with many studies reporting null results and finding no relationship between neurovascular measures and behavioral outcomes. The lack of consensus in the prior literature may have to do with typically small sample sizes and varying times post stroke, as at different stages of recovery different mechanisms might be at play. In some of the studies reviewed above (e.g., Fridriksson et al., 2012; Boukrina et al., 2019), the inclusion of individuals with both ischemic and hemorrhagic strokes might have obscured structure-function relationships, as changes in blood flow patterns are likely to be more pronounced in instances of ischemic stroke. Other methodological issues, such as nonuniform and substandard imaging sequences and processing algorithms, arbitrary cutoffs, variable delineation of perilesional areas, and different parcellations, might also be contributing to inconclusive and contradictory findings. The limited use of CVR measures also precludes a comprehensive understanding of recovery mechanisms. Larger group studies in both acute, subacute, and chronic post stroke aphasia are needed to address the existing knowledge gaps. Future studies could benefit from using advanced perfusion and CVR sequences that incorporate the latest recommendations on acquisition and processing of neurovascular metrics (Stickland et al., 2021; Lindner et al., 2023). Beyond implementation of a more refined methodological approach and systematic data processing algorithms, we foresee several important and promising avenues for future work in this area.

First, as outlined in detail in the previous sections, CBF is a static measure of blood flow whereas CVR is a dynamic measure of vascular reactivity. These two approaches can complement each other in outlining a comprehensive picture of the functionality and health of the remaining tissue post stroke. As noted above, it has been shown that baseline CBF in healthy individuals is positively correlated with CVR, meaning that regions with higher perfusion are more likely to show an increased CVR response (Stickland et al., 2022). If CBF measurements show that certain language regions increase perfusion post stroke, they could possibly indicate a higher CVR response and collateral flow or vice versa. However, understanding the uncoupling of CBF and CVR is also important for revealing the vascular pathology in the remaining tissue. For example, in cases of high arterial stiffness, the two quantities are uncoupled with certain regions showing lower CBF and preserved CVR (Jefferson et al., 2018). In the end, both metrics are needed to fully understand brain health and tissue functionality. Future neurovascular studies would greatly benefit from using a combination of CBF and CVR measures that could generate novel insights into the mechanisms of post stroke language deficits.

Second, the vast majority of prior neurovascular studies in stroke have been cross-sectional, with a limited number of treatment studies looking at measures of blood flow at multiple time points. As pointed out in the reviewed literature, blood flow post stroke is likely to change from the acute to the chronic stages. It is important that future work carefully outlines these changes and their relationship to behavioral improvements at different times post stroke. Longitudinal studies of blood flow are needed to comprehensively understand post stroke aphasia recovery.

Third, one of the outstanding issues in the stroke perfusion literature is untangling decreases in blood flow from delayed blood flow. Lowered CBF following stroke might be due to lower blood flow or slower dynamics or possibly both. Slowed blood flow dynamics alone may impact processing capacity, as blood may not be delivered to brain regions in time to support rapid language processing. Previously a number of functional MRI studies demonstrated delayed hemodynamic response function in different perisylvian areas of the left hemisphere in individuals with chronic aphasia (Fridriksson et al., 2006; Bonakdarpour et al., 2007). Measures of ATTs that index the time it takes blood to arrive in a given area can help to directly address these questions. Thus, future studies should implement advanced multi-PLD perfusion sequences as from them both measures of CBF and ATTs can be derived, reflecting perfusion and blood flow dynamics, respectively. Additional promising methods have emerged that can measure long time delays (or lags) of the blood-born signal to arrive in each brain region. They are based on recursive cross-correlation of the low frequency component of resting-state fMRI BOLD signals with a global regressor (Tong and Frederick, 2014) and these have shown great success in cases such as Moyamoya disease where the ATT might beyond what standard ASL sequences can capture (Donahue et al., 2016). Ultimately, adding measures of blood flow dynamics will help to explain the nature of decreased perfusion post stroke.

Finally, to outline the mechanisms of language recovery post stroke, it is crucial to examine disrupted neurovascular coupling and the interactions between changes in neuronal activity and blood flow. The available findings suggest that adequate perfusion of tissue in the regions that support linguistic processing is critical for upregulating neural activity and promoting language recovery (Thompson et al., 2010, 2017; Fridriksson et al., 2012), with lower perfusion possibly limiting each region’s contribution to language recovery (Ivanova et al., in press). However, a complete picture of language recovery needs to consider both components of the neuro-vascular system, i.e., how the brain’s vasculature dynamically interacts with neural mechanisms to assure brain function (Bundo et al., 2002; Girouard and Iadecola, 2006). While it may be that adequate perfusion levels are a prerequisite to restoration of functions (Thompson et al., 2010; Fridriksson et al., 2012; Boukrina et al., 2019), it is possible that neural activity itself drives restoration of normal blood flow levels. That is, repeated activation of a region might lead to changes in blood supply that eventually restore blood flow in that region to premorbid levels.

Changes in functional activation in individuals with aphasia relative to healthy participants during a language fMRI task have been observed in a number of studies. A large metanalysis of fMRI studies in aphasia showed that increased activation in left hemisphere language regions and possibly temporal areas in the right hemisphere were associated with greater residual language ability (Wilson and Schneck, 2021). Stroke can also have an indirect effect in remote regions that are functionally connected with the damaged regions resulting in changes in functional connectivity. Siegel and colleagues found that disruption in functional connectivity was related to behavioral outcomes including language and that recovery of functional connectivity was associated with behavioral improvements (Siegel et al., 2016, 2018). However, it is noted that measures of BOLD-based functional activation and functional connectivity measures relate to blood flow measures via the mechanisms of neurovascular coupling. In stroke, observed changes in functional activation or connectivity need to be taken into consideration in conjunction with the observed CBF or CVR measures as the neurovascular coupling might be disrupted (He et al., 2020). This can be done statistically by including baseline CBF/CVR alongside measured activity/connectivity measures in predictive models, or by directly measuring the hemodynamic effect and using this to correct these measures. For the latter, one way is to extract lags, wherein the CBF or BOLD responses are measured relative to a global signal signifying how much vascular delay each part of the brain has relative to the whole brain mean (similar measures can be extracted using CVR; Stickland et al., 2022). This metric is a proxy for hemodynamic delays (Tong et al., 2017) and it has been used in stroke studies to correct functional connectivity values (Siegel et al., 2016). Finally, in the context of TMS treatment, CBF and CVR can be used to identify individual differences in TMS responses. In a study by Gratton et al. (2014), the authors found that in healthy participants TMS increased CBF in the stimulated region and that these changes in blood flow were related to the intrinsic functional connectivity of that region in each individual. Beyond local changes, TMS can also affect distant regions via connections between the target area and the rest of the brain. For instance, one study showed that TMS affected functional connectivity of regions proximal to the stimulation site as well as distant brain regions that were functionally connected to the site of stimulation (Castrillon et al., 2020). By combining the results of these studies, one can argue that subject specific TMS targets can be selected based on their CBF/CVR values as well as their connectivity profile.

In all, the complex relationship between neuronal activity and blood flow needs to be explored via multimodal neuroimaging techniques in future longitudinal recovery studies. Specifically, incorporating CVR measures alongside CBF measures can inform the neurovascular profile of different regions, and when used alongside functional MRI measures, it can confirm that observed changes in the BOLD signal truly reflect changes in level of neural activity/connectivity (Geranmayeh et al., 2015). Further, neurovascular measures can be used not only to understand spontaneous and treatment-induced recovery but also to facilitate selection of stimulation sites where candidate regions can be selected based on their neurovascular profile. These are promising avenues for future studies that will require the collection of multimodal neuroimaging data and well-controlled experimental designs.

Stroke is a disorder of blood flow and yet blood flow metrics have been largely neglected in recovery studies. Recent methodological advances in both perfusion and CVR imaging are making these measures more accessible to researchers and at the same time more sensitive to alterations observed in stroke and possible changes in recovery. In our future work, we hope to follow these promising avenues of research and strongly encourage others to begin including neurovascular measures in their neuroimaging studies of aphasia.
